# Specific Missense Alleles of the Arabidopsis Jasmonic Acid Co-Receptor COI1 Regulate Innate Immune Receptor Accumulation and Function

**DOI:** 10.1371/journal.pgen.1003018

**Published:** 2012-10-18

**Authors:** Yijian He, Eui-Hwan Chung, David A. Hubert, Pablo Tornero, Jeffery L. Dangl

**Affiliations:** 1 Department of Biology, The University of North Carolina at Chapel Hill, Chapel Hill, North Carolina, United States of America; 2 Howard Hughes Medical Institute, The University of North Carolina at Chapel Hill, Chapel Hill, North Carolina, United States of America; 3 Curriculum in Genetics and Molecular Biology, The University of North Carolina at Chapel Hill, Chapel Hill, North Carolina, United States of America; 4 Department of Microbiology and Immunology, The University of North Carolina at Chapel Hill, Chapel Hill, North Carolina, United States of America; 5 Carolina Center for Genome Sciences, The University of North Carolina at Chapel Hill, Chapel Hill, North Carolina, United States of America; Virginia Tech, United States of America

## Abstract

Plants utilize proteins containing nucleotide binding site (NB) and leucine-rich repeat (LRR) domains as intracellular innate immune receptors to recognize pathogens and initiate defense responses. Since mis-activation of defense responses can lead to tissue damage and even developmental arrest, proper regulation of NB–LRR protein signaling is critical. RAR1, SGT1, and HSP90 act as regulatory chaperones of pre-activation NB–LRR steady-state proteins. We extended our analysis of mutants derived from a *rar1* suppressor screen and present two allelic *rar1 suppressor* (*rsp*) mutations of Arabidopsis *COI1*. Like all other *coi1* mutations, *coi1^rsp^* missense mutations impair Jasmonic Acid (JA) signaling resulting in JA–insensitivity. However, unlike previously identified *coi1* alleles, both *coi1^rsp^* alleles lack a male sterile phenotype. The *coi1^rsp^* mutants express two sets of disease resistance phenotypes. The first, also observed in *coi1-1* null allele, includes enhanced basal defense against the virulent bacterial pathogen *Pto* DC3000 and enhanced effector-triggered immunity (ETI) mediated by the NB–LRR RPM1 protein in both *rar1* and wild-type backgrounds. These enhanced disease resistance phenotypes depend on the JA signaling function of COI1. Additionally, the *coi1^rsp^* mutants showed a unique inability to properly regulate RPM1 accumulation and HR, exhibited increased RPM1 levels in *rar1*, and weakened *RPM1*-mediated HR in *RAR1*. Importantly, there was no change in the steady-state levels or HR function of RPM1 in *coi1-1*. These results suggest that the coi1^rsp^ proteins regulate NB–LRR protein accumulation independent of JA signaling. Based on the phenotypic similarities and genetic interactions among *coi1^rsp^*, *sgt1b*, and *hsp90.2^rsp^* mutants, our data suggest that COI1 affects NB–LRR accumulation via two NB–LRR co-chaperones, SGT1b and HSP90. Together, our data demonstrate a role for COI1 in disease resistance independent of JA signaling and provide a molecular link between the JA and NB–LRR signaling pathways.

## Author Summary

To detect pathogen attack and subsequently trigger defense responses, plants utilize immune receptors composed of a nucleotide binding site (NB) domain and a C-terminal leucine-rich repeat (LRR) domain that function inside the cell. To identify regulators of NB–LRR protein accumulation and activity, we performed a genetic screen in the model plant *Arabidopsis thaliana* to isolate mutants that affect NB–LRR protein accumulation levels and NB–LRR triggered disease resistance. Here, we introduce two mutant alleles of *COI1*, a gene which encodes a well-characterized receptor for the phytohormone Jasmonic Acid (JA). It is widely accepted that COI1 is involved in JA signaling-dependent disease resistance. However, our new *coi1* mutants affected NB–LRR accumulation in a manner independent of the JA signaling pathway. This indicated that not all disease resistance effects of COI1 require JA signaling. We also observed a link between COI1 and the RAR1-SGT1b-HSP90 co-chaperone complex, which plays a critical role in regulation of NB–LRR protein accumulations.

## Introduction

During their life cycle, plants have to fend off microbial pathogens including fungi, bacteria, viruses, and nematodes. To protect themselves, plants rely on the innate immune system of each plant cell to detect pathogen attack and subsequently activate disease resistance responses. The plant immune system relies on two inter-related branches. The first branch utilizes pattern recognition receptors (PRRs) to identify conserved pathogen associated molecular patterns (PAMPs). This recognition then initiates PAMP-triggered immunity (PTI) [1]–[3]. Although PTI can restrict further colonization in some cases, successful pathogens are still able to evade or suppress PTI with their effectors [4]. These proteins contribute to pathogen virulence by interfering with various plant defense-related cellular processes. However, effectors can also be recognized by the intracellular NB–LRR receptor proteins of the plant innate immune system [5]. Recognition of effectors results in effector-triggered immunity (ETI) and is the second branch of the plant immune system [1]–[3]. NB–LRR proteins contain a centrally located nucleotide binding site (NB) domain and a C-terminal leucine-rich repeat (LRR) domain. Mammalian NB–LRR containing (NLR) proteins mediate analogous processes in mammalian innate immunity [6].

NB–LRR-mediated ETI is typically associated with a form of programmed cell death at the infection site termed the hypersensitive response (HR) [1]–[3]. If not controlled, this strong response can lead to unnecessary tissue damage. Proper regulation of HR and therefore appropriate regulation of pre-activation, resting state NB–LRR proteins is critical [7]–[9]. Genetic analyses uncovered three genes, *RAR1*, *SGT1* and *HSP90*, as key regulators of NB–LRR stability and activity [10]–[18]. RAR1, SGT1 and HSP90 proteins can interact independently with one another [13], [14], [16], and can cooperate as a molecular chaperone complex to regulate NB–LRR stability and function. HSP90 is usually thought to be the central subunit of the complex [19], [20]. RAR1 affects the conformational dynamics of HSP90, and modulates the “lid-open” conformation required for loading client NB–LRR proteins [21], [22]. However, the functional mechanism by which the RAR1-SGT1-HSP90 complex maintains NB–LRR levels remains poorly understood.

As highly conserved proteins, SGT1 and HSP90 also interact with each other in mammalian cells, and play essential roles in mammalian immune responses mediated by NLR proteins. By co-immunoprecipitation experiments, both SGT1 and HSP90 were found to associate with many NLR proteins including NOD1 (Nucleotide-binding Oligomerization Domain 1), NOD2 (Nucleotide-binding Oligomerization Domain 2), and NALP3 (NACHT, LRR and PYD domains-containing Protein 3) [23], [24]. In mammalian cells, treatment with geldanamycin (GDA), a chemical inhibitor of HSP90, impaired NOD2-induced NF-κB activity and NALP3-mediated inflammatory responses [24]. Knockdown of HSP90 by RNAi or GDA treatment also reduced the accumulation levels of NOD1 and NOD2 [23]. These results demonstrated that mammalian HSP90 is required for both NLR stability and function. In contrast, mammalian SGT1 is only required for NLR functions such as NOD1-mediated cytokine production, NOD1-mediated cell death, and NALP3-mediated inflammatory responses, but not for NLR stability [23], [24]. Plant SGT1, however, functions in both NB–LRR activity and stability [25]. Moreover, mammalian SGT1 knockdown reduced the association between HSP90 and the NALP3 LRR domain, indicating that mammalian SGT1 functions as a co-chaperone of mammalian HSP90 to regulate client NLR protein [24]. Unlike plant RAR1, CHP1 (CHORD-containing Protein 1), a homolog of RAR1 in mammals, is not involved in regulating NLR protein accumulation or function [24]. Taken together, the SGT1-HSP90 chaperone complex has functions for mammalian NLR protein stability and activity, analogous to its functions for plant NB–LRR biology [19], [20].

During infection, both host plants and pathogens regulate phytohormone signaling to enhance their defense and virulence respectively. Jasmonic Acid (JA) controls a well characterized example of phytohormone signaling required for both disease resistance and effector-induced susceptibility that is an outcome of the suppression of PTI [26], [27]. The JA receptor, *COI1*, is the key regulator of JA signaling [28]–[31]. Mutations in *COI1* cause defects in JA responses and reproductive development [32], [33]. Of note, mutations in *COI1* also affect, negatively or positively, disease resistance against various plant pathogens [29], [33]–[41].

*COI1* encodes an F-box protein that is a component of the SCF^COI1^ (Skp1/Cullin/F-box^COI1^) E3 ubiquitin ligase complex [31], [32], [42]. The function of COI1 is to specifically bind target proteins to promote ubiquitination and degradation by the 26S proteasome [31]. It is therefore assumed that COI1 regulates JA signaling and disease resistance via degradation of specific proteins. The connection between JA signaling and SCF^COI1^-mediated protein degradation has been confirmed. The JASMONATE ZIM DOMAIN (JAZ) family proteins act as repressors of MYC2, a key transcriptional activator of JA responses, by directly interacting with MYC2. JA-Ile, a bioactive JA conjugate, induces the degradation of JAZ proteins by enhancing the protein interaction between JAZs and COI1, and thus de-represses JA-related transcription activation [28], [29], [31], [43]. The JAZ and MYC proteins also play a role in disease resistance. Overexpression of JAZ1Δ3A, a C-terminal deletion form of JAZ1, led to enhanced disease resistance against *Pto* DC3000 in Arabidopsis [29]. The triple mutant for transcription factor genes *MYC2*, *MYC3*, and *MYC4*, which are all repressed by JAZ proteins, was as resistant against *Pto* DC3000 as the *coi1* mutant [44].

In this study, we extend our previously described suppressor screen for new mutants that recover impaired *RPS5* function in *rar1* [21]. We introduce two novel missense alleles of *COI1* that suppress the disease resistance phenotypes associated with *rar1* mutation. Surprisingly, these two *coi1 rar1 suppressor* (*rsp*) alleles are completely fertility, in contrast to the male sterility associated with all other *coi1* mutant alleles [32], [43], [45]. Like *sgt1b* and the *hsp90.2^rsp^* alleles [21], these two *coi1^rsp^* alleles interact with *rar1* to restore the disease resistance responses mediated by some NB–LRRs and the accumulation of at least RPM1. Moreover, we demonstrate that overexpression of *SGT1b* can partially inhibit the *coi1^rsp^*-enhanced accumulation of RPM1 and *RPM1*-mediated disease resistance in *rar1*. We also observe non-allelic non-complementation, a rare genetic interaction, between *coi1^rsp^* mutants and *hsp90.2-7^rsp^* mutant. These results support the hypothesis that coi1^rsp^ proteins regulate NB–LRR levels via SGT1b and HSP90.

## Results

### Identification of new alleles of *COI1* and of the *rsp3* mutant

**Figure 1:**
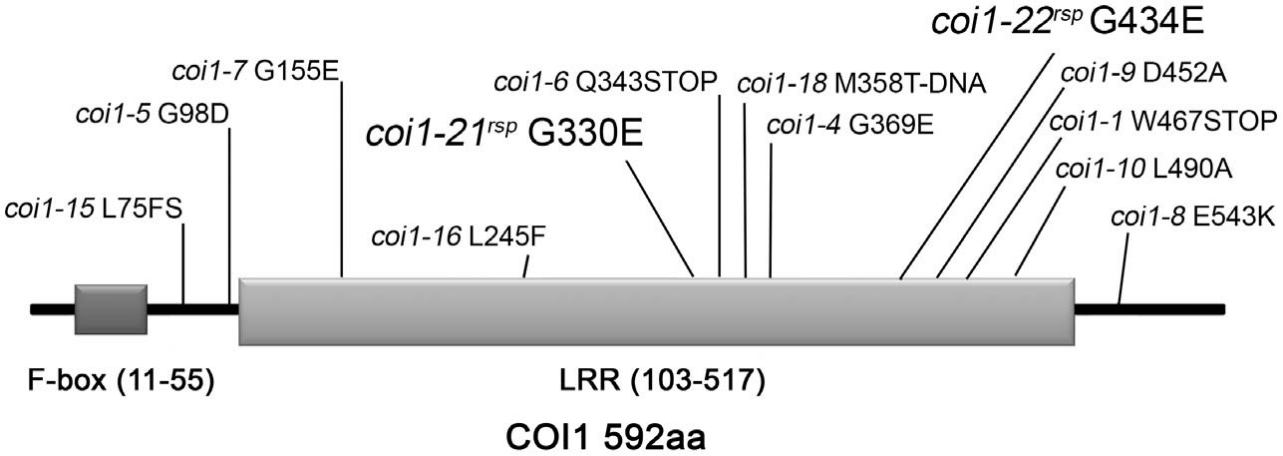
Mutations identified in COI1. The F-box domain and the LRR domain are shown in dark and light gray, respectively. The allele designation and associated amino acid change is shown in relation to its linear position. New alleles introduced in this paper are shown with larger font.

**Figure 2:**
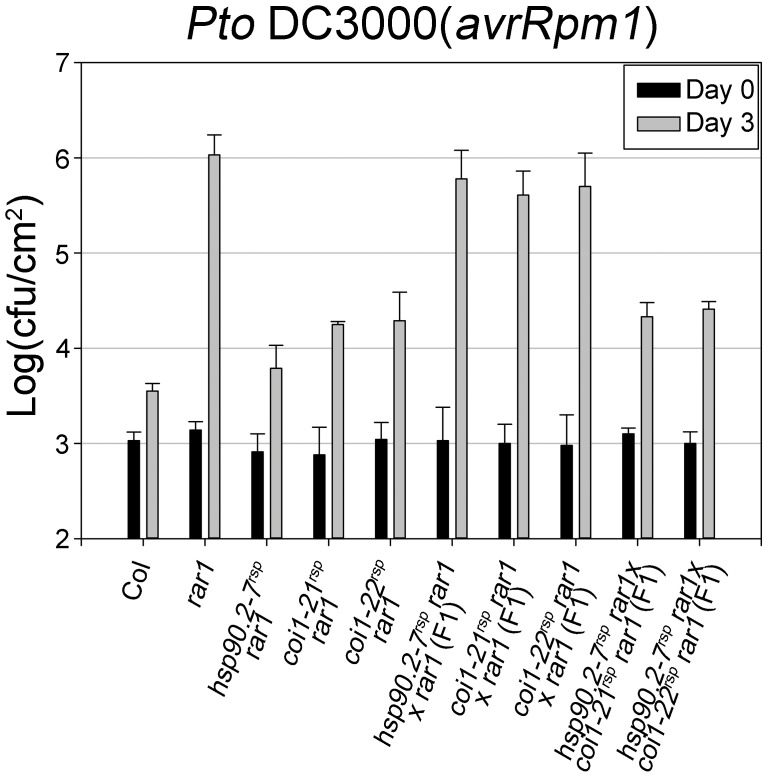
*COI1* and *HSP90* interact genetically to regulate disease resistance. Bacteria *Pto* DC3000(*avrRpm1*) were hand-infiltrated into leaves of each indicated genotype and counted at day 0 and day 3. Error bars represent 2× SE. The result displayed is one of two independent analyses giving similar results.

To identify new genes that act with *RAR1* to regulate NB–LRR accumulation and activation, we performed a suppressor screen for new mutants which can suppress the disease susceptibility observed in *rar1-21* (a stop mutation in Q52) [21]. Five *rar1 suppressor* (*rsp*) mutants were identified from approximately 200,000 M2 plants from 50 M2 pools that recover resistance responses to both *Pto* DC3000(*avrPphB*) and *Pto* DC3000(*avrRpm1*) [21]. Based on map-based cloning and subsequent allele sequencing, two of the five mutants were found to have mutations in *COI1* (At2g39940). To follow accepted nomenclature conventions, we designated these two mutant alleles, *coi1-21^rsp^* and *coi1-22^rsp^*, respectively (Figure 1). Based on disease symptoms after inoculation of *Pto* DC3000(*avrRpm1*) on backcross F1 and F2 populations, both of the *coi1^rsp^* mutants were completely recessive (Table S1). This conclusion was also confirmed by growth assays of *Pto* DC3000(*avrRpm1*) in backcross F1 plants (Figure 2). The *coi1-21^rsp^* mutation is a G/A transition which leads to a G330E missense change in the COI1 protein. The *coi1-22^rsp^* mutation is a G/A transition resulting in a G434E missense change in the protein. Both mutations are within conserved LRR domains (Figure 1). Using the crystal structure of the Arabidopsis COI1 protein, we observed that neither coi1^rsp^ mutation is localized in the interfaces of COI1 that make up the ASK1-binding region and the ligand-binding pocket [31].

In addition, another *rar1 suppressor* (*rsp*) mutant called *rsp3* was isolated from this screen. *rsp3* suppressed all known *rar1* phenotypes, and was localized in a 7 Mbp region on chromosome I (Figure S1). A single allele, dominant mutation was identified in *rsp3*; its detailed characterization is beyond the scope of this work.

### *COI1* and *HSP90* interact genetically to regulate disease resistance

The disease resistance restoration phenotypes of *hsp90.2-7^rsp^* and either *coi1^rsp^* alleles in *rar1* are fully recessive with respect to their respective wild type phenotypes ([21], Figure 2). We monitored *in planta* growth of *Pto* DC3000(*avrRpm1*) to measure *RPM1*-mediated disease resistance in F1 plants of *hsp90.2-7^rsp^*×*coi1^rsp^* crosses (Figure 2). The resulting F1 plants were as resistant to *Pto* DC3000(*avrRpm1*) as their parental *coi1^rsp^* plants. We also tested F1 plants of crosses between *hsp90.2-7^rsp^* and either *coi1^rsp^* allele for disease symptoms after inoculation of *Pto* DC3000(*avrRpm1*). The F1 plants displayed resistance against *Pto* DC3000(*avrRpm1*) (Table S2). In addition, we observed that a part of the F2 progenies from each F1 were susceptible to *Pto* DC3000(*avrRpm1*) (Table S2). These results clearly demonstrate non-allelic non-complementation between *hsp90.2-7^rsp^* and *coi1^rsp^* mutants, suggesting that the two proteins function in the same process, and likely do so in physical proximity [14], [46], [47].

### *coi1^rsp^* alleles and *coi1-16* partially suppress known *rar1* phenotypes

**Figure 3:**
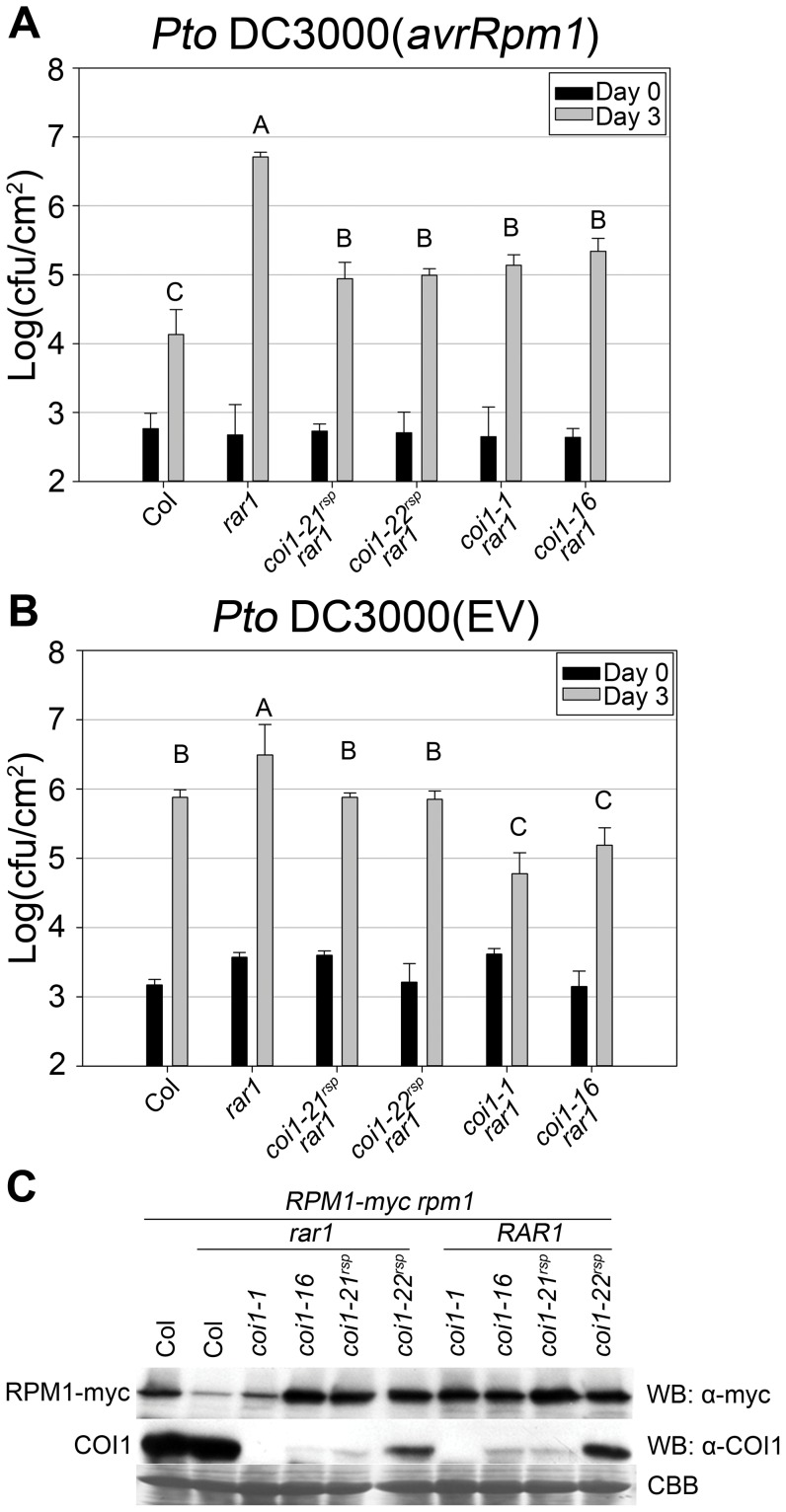
*coi1^rsp^* mutants suppress *rar1* phenotypes and are not null allele. (A–B) Bacterial growth analysis of (A) *Pto* DC3000(*avrRpm1*) and (B) *Pto* DC3000(EV). Bacteria were hand-infiltrated into leaves of each indicated genotype and counted at day 0 and day 3. Error bars represent 2× SE. Pairwise comparisons for all means for bacterial growth on day 3 were performed with One-Way ANOVA test followed by Tukey-Kramer HSD at 95% confidence limits. (C) Western blot analysis of RPM1-myc and COI1 levels in the indicated genotypes. RuBisCo levels stained by Coomassie Brilliant Blue serve as loading controls. The pathogen growth assays were performed independently three times with similar results. The western blots were performed independently two times with similar results. Both RPM1-myc and COI1 blots used the same protein samples.

*hsp90.2^rsp^* alleles isolated from our *rar1* suppressor screen recover all known defective NB–LRR functions in a *rar1* mutant background [21]. However, a previously published *rar1* suppressor mutant, *sgt1b*, only affected a limited number of NB–LRR protein functions [25]. We therefore tested both *coi1^rsp^* alleles to determine whether they have any NB–LRR specificity in their suppression of *rar1*. The *coi1^rsp^* alleles partially suppress *rar1* for *RPS5* and *RPM1* functions, and fully suppress *rar1* for *RPS2* function (Figure 2, Figure S2A and S2B, Figure 3A).

*rar1* exhibits enhanced disease susceptibility to the virulent bacterial strain *Pto* DC3000(EV) [18], [21], [25]. This phenotype might be due to a *RAR1* function in basal defense, for example an additive effect of globally lowered accumulation of multiple NB–LRR proteins [1]–[3]. As measured by inhibition of bacterial growth, both *coi1^rsp^* alleles completely suppressed the enhanced disease susceptibility phenotype in *rar1* (Figure 3B).

NB–LRR activation can trigger the hypersensitive response (HR) as well as disease resistance responses. *RAR1* is required for HR mediated by many NB–LRR proteins. *sgt1b* is able to suppress the loss of *RPS5*-mediated disease resistance in a *rar1* mutant, but not the loss of *RPS5*-mediated HR [25]. To test if NB–LRR-dependent HR is also recovered in *coi1^rsp^ rar1* double mutants, we measured ion leakage as a proxy for HR to quantify *RPM1*-mediated HR in plants. Notably, the *coi1^rsp^* alleles did not suppress *rar1* for impaired *RPM1*-triggered HR (Figure S2C). However, the *coi1^rsp^ rar1* plants did recover *RPM1*-mediated disease resistance, measured via pathogen growth restriction (Figure 3A).

RPS5, RPM1 and RPS2 all belong to the CC-NB–LRR subclass. The functions of some TIR-NB–LRR proteins also require RAR1. The effect of *coi1^rsp^* on TIR-NB–LRR function was tested using the pathogenic oomycete *Hyaloperonospora arabidopsidis* (*Hpa*) isolate Emwa1 to trigger *RAR1*-dependent *RPP4*-mediated disease resistance [48]. Neither of the two *coi1^rsp^ rar1* double mutants inhibited the growth of Emwa1 (Figure S2D). This indicates that *RPP4* function is not recovered in *rar1* in the presence of either *coi1^rsp^* allele. Thus, the *coi1^rsp^* alleles possibly suppress *rar1* only for CC-NB–LRR functions.

The accumulation of all tested NB–LRR proteins is reduced in *rar1* plants, implying that the biochemical function of RAR1 is to maintain the stability of NB–LRR proteins [7], [18], [21], [25], [49]. We wondered whether *coi1^rsp^* alleles could suppress the decrease of NB–LRR protein accumulation in *rar1*. We introduced our transgenic, myc-tagged *RPM1* [50] into the *coi1^rsp^ rar1* mutants by crossing and marker-assisted selection. The *coi1^rsp^* alleles suppressed the lowered RPM1-myc accumulation in *rar1* (Figure 3C). Hence, the *coi1^rsp^* alleles suppress the biochemical phenotype of *rar1*.

The *coi1^rsp^* alleles are phenotypically different from two reference alleles, *coi1-1* (a protein null (encoding W467STOP [32]; Figure 1) and *coi1-16* (encoding L245F [45]; Figure 1), which are also completely or conditionally male sterile. We therefore tested whether either *coi1-1* or *coi1-16* could suppress *rar1*. Similar to the *coi1^rsp^* alleles, *coi1-1* and *coi1-16* enhanced disease resistance responses against both *Pto* DC3000(*avrRpm1*) and *Pto* DC3000(EV) in a *rar1* background (Figure 3A, 3B). The increase in disease resistance against *Pto* DC3000(EV) was even higher than that caused by the *coi1^rsp^* alleles (Figure 3B). To our surprise, *coi1-16* resulted in the recovery of RPM1-myc accumulation in *rar1*, but *coi1-1* did not (Figure 3C). However, *coi1-16* and *coi1-1* express equivalent enhanced disease resistance in *rar1*. Thus, the “restoration” of disease resistance responses against *Pto* DC3000(*avrRpm1*) that we observed in *coi1-1 rar1* is not due to restoration of NB–LRR protein levels, but rather to bypass suppression of *rar1* disease susceptibility. This is likely caused by enhanced basal defense possibly related to the antagonistic relationship between JA- and SA-dependent signaling (Figure 3). The growth of *Pto* DC3000(*avrRpm1*) and *Pto* DC3000(EV) at 3 dpi was about the same in *coi1-16 rar1* plants (Figure 3). Thus, the restored disease resistance in *coi1-16 rar1* is likely due to enhanced basal defense, not RPM1 function, although there is a restoration of RPM1-myc accumulation in *coi1-16 rar1*.

Since the *coi1-1* null allele cannot suppress *rar1*, we suggest that the *coi1^rsp^* alleles and *coi1-16* are recessive gain-of-function alleles for the *rar1* suppression phenotypes. They are also loss-of-function alleles for the JA response phenotypes as detailed below.

### The *coi1^rsp^* mutations negatively regulate *RPM1*-dependent HR in otherwise wild-type plants

**Figure 4:**
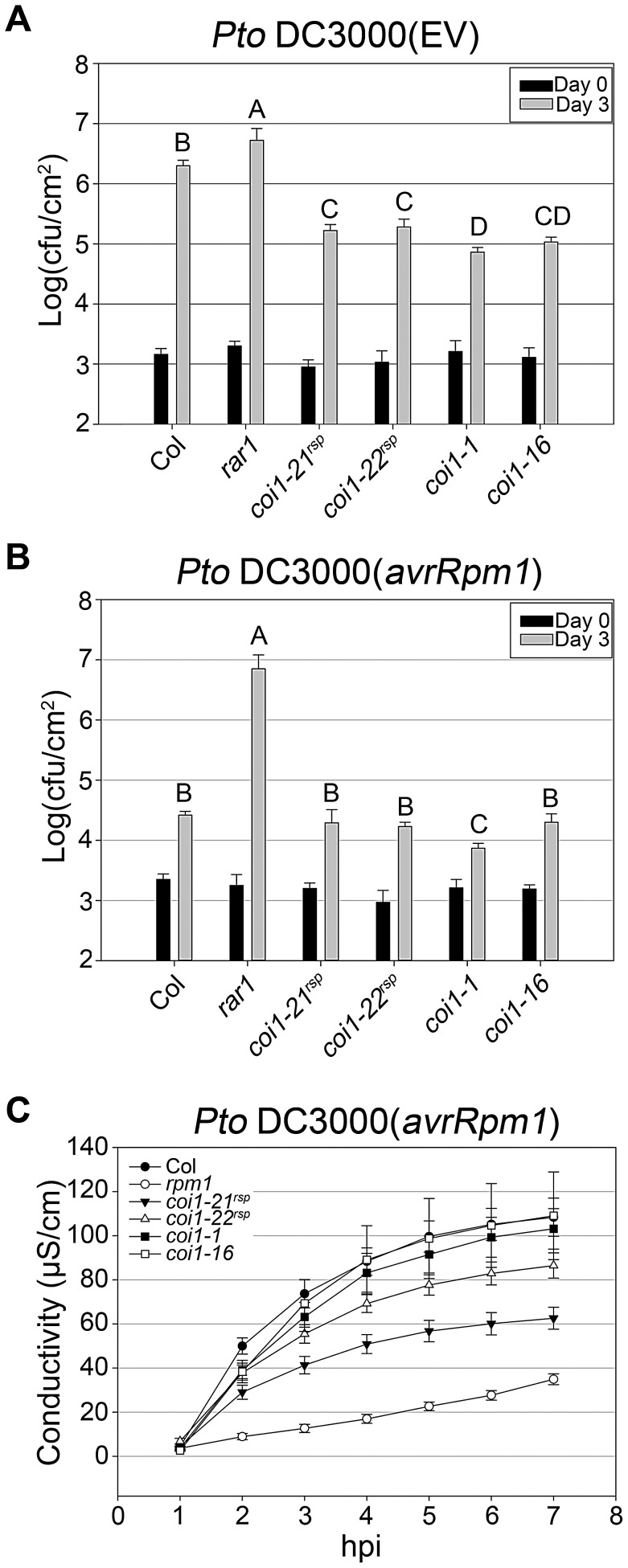
*coi1^rsp^* alleles exhibit enhanced basal defense and additionally weakly suppress RPM1 HR function. (A–B) Bacterial growth analysis of *Pto* DC3000(EV) (A) and *Pto* DC3000(*avrRpm1*) (B). Bacteria were hand-infiltrated into leaves of each indicated genotype and counted at day 0 and day 3. Error bars represent 2× SE. Pairwise comparisons for all means for bacterial growth on day 3 were performed with One-Way ANOVA test followed by Tukey-Kramer HSD at 95% confidence limits. (C) Conductivity measurements after inoculation with high concentration *Pto* DC3000(*avrRpm1*) (5×10^7^ cfu/ml). Error bars represent 2× SE. The pathogen growth and HR assays were performed independently a minimum of three times with similar results.

We introduced the *coi1^rsp^* alleles into an isogenic *RAR1* background using marker-assisted breeding (see Methods). To further study the role of COI1 in regulating RPM1 function, we inoculated both *coi1^rsp^* alleles, *coi1-1* and *coi1-16* plants with *Pto* DC3000(*avrRpm1*) and measured bacterial growth (Figure 4B). The *coi1^rsp^* and *coi1-16* mutants were as resistant as wild type. The *coi1-1* mutant displayed slightly enhanced resistance compared with wild type. We also measured *RPM1*-mediated HR in these *coi1* single mutants using the ion leakage assay (Figure 4C). Surprisingly, both *coi1^rsp^* alleles weakly suppressed *RPM1*-mediated HR. We crossed *RPM1-myc* into these *coi1^rsp^*, *coi1-1* and *coi1-1*6 single mutants and measured RPM1-myc protein levels (Figure 3C). We observed no obvious changes in RPM1-myc levels in any of the single *coi1* mutant. We conclude from these data that *coi1^rsp^* mutations differentially regulate RPM1 function in *rar1* or *RAR1* backgrounds.

### Increased RPM1 accumulation in *coi1^rsp^* and *coi1-16* is post-transcriptionally regulated

Loss of *COI1* leads to elevated levels of salicylic acid (SA) in plants [37], and elevated SA levels can induce the expression of some *NB–LRR*-encoding genes [51]–[53]. *NB–LRR* expression is not changed in *rar1* (Figure S4, [49]). We measured *RPM1* mRNA levels in the *coi1^rsp^*, *coi1-1*, and *coi1-16* mutant plants in the context of wild-type *RAR1* by RT-qPCR in order to determine whether the increased RPM1-myc protein levels noted in *coi1^rsp^* and *coi1-16* were due to enhanced transcription. Wild type and *rar1* plants were used as controls. We detected no enhancement of *RPM1* mRNA levels among the tested *coi1* mutants (Figure S4), indicating that the *coi1^rsp^* and *coi1-16* alleles restore RPM1 protein levels by a post-transcriptional mechanism in *rar1*.

### The *coi1^rsp^* alleles are JA–insensitive

**Figure 5:**
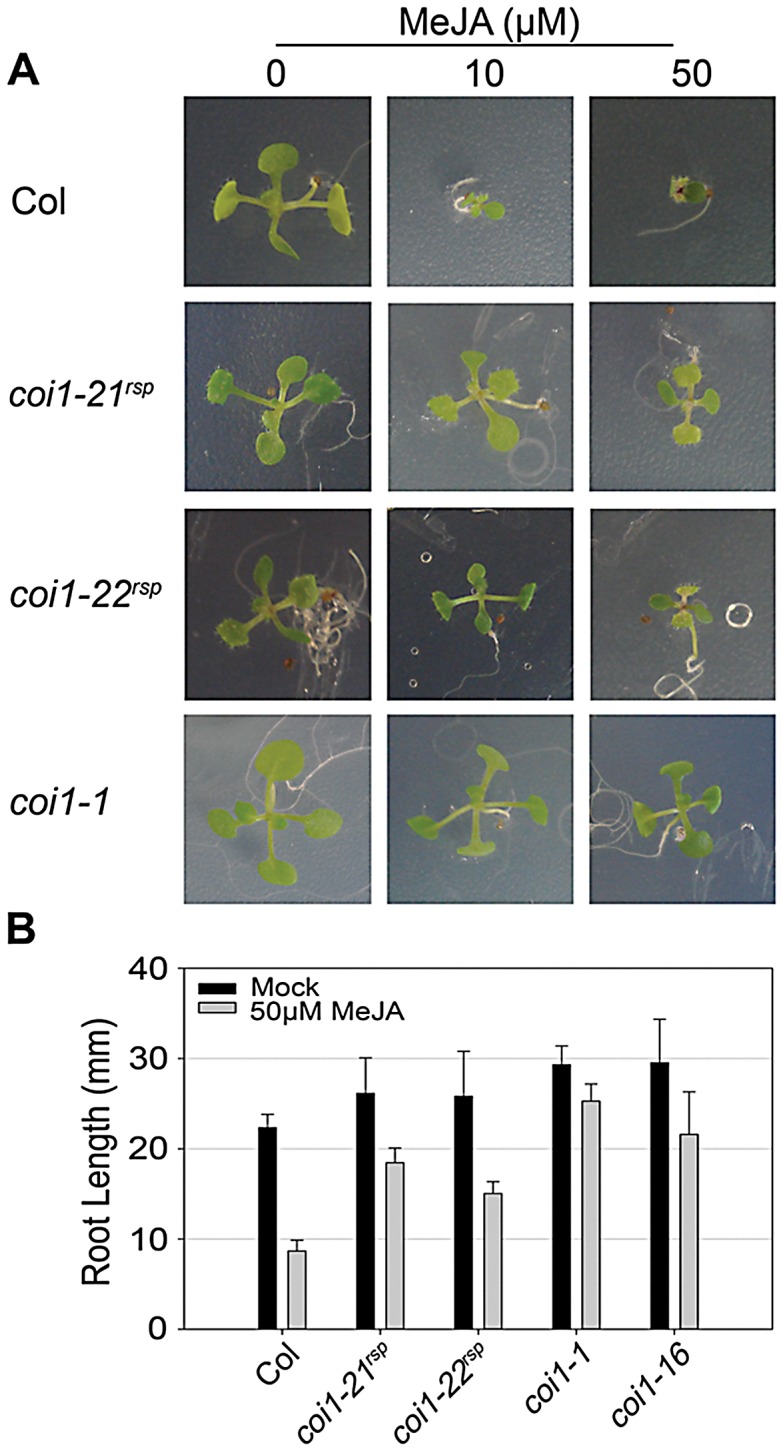
*coi1^rsp^* alleles are insensitive to JA. (A) Seedlings of the indicated genotypes were grown on MS medium (control) or medium containing 10 or 50 µM MeJA. (B) Inhibition of root elongation by 50 µM MeJA in at least twenty seedlings of indicated genotypes. This assay was performed independently three times with similar results.

COI1 has an essential role in JA signaling; all previously isolated *COI1* mutations caused insensitivity to JA-mediated inhibition of seedling growth [32], [43], [45]. We compared JA-insensitivity phenotypes of the *coi1^rsp^* alleles to *coi1-1* using a growth inhibition assay where plants were grown in the presence of MeJA, a functional JA derivative (Figure 5). Like *coi1-1*, the MeJA-treated *coi1^rsp^* seedlings grew on MeJA-containing media, while the growth of wild type seedlings was severely inhibited (Figure 5A). MeJA treated *coi1^rsp^* seedlings were clearly smaller than the untreated seedlings, suggesting that the *coi1^rsp^* alleles are not as insensitive to JA as *coi1-1*. We quantified these phenotypes with a root elongation assay (Figure 5B). The null allele *coi1-1* displayed root growth inhibition of only about 14% in the presence of 50 µM MeJA. Compared with *coi1-1*, *coi1-16* and both *coi1^rsp^* alleles displayed intermediate insensitivity to MeJA treatment. Their root growth was inhibited about 27%, 30% and 42% respectively, while the root growth inhibition was more than 60% in wild type seedlings. Thus, the *coi1^rsp^* alleles are JA-insensitive.

JA signaling is important in disease resistance responses. *coi1* and other JA insensitive mutants exhibit enhanced resistance to the virulent bacterial strain *Pto* DC3000(EV) [29], [37], [38]. We measured the growth of *Pto* DC3000(EV) in our *coi1^rsp^* alleles, *coi1-1*, and *coi1-16* (Figure 4A). The *coi1^rsp^* alleles also displayed enhanced resistance to *Pto* DC3000(EV), although the increase in the *coi1^rsp^* alleles was slightly lower than in the reference alleles *coi1-1* and *coi1-16*.

### *coi1^rsp^* alleles are not null alleles

The *coi1^rsp^* alleles are quantitatively different than the *coi1-1* null allele with respect to JA responses (Figure 5B) and enhanced resistance to *Pto* DC3000(EV) (Figure 4A). We noted decreased COI1 protein accumulation levels in *coi1-21^rsp^*, *coi1-22^rsp^* and *coi1-16* plants compared to wild type and *rar1* plants (Figure 3C). As expected, no detectable amount of COI1 protein was observed in *coi1-1*. The residual accumulations of COI1 protein confirmed that the *coi1^rsp^* alleles and *coi1-16* are not COI1 null alleles.

### The *sgt1b* mutant is insensitive to JA responses

To determine whether other NB–LRR regulators function in regulating JA responses, we tested the JA response in the mutants of three NB–LRR co-chaperones, *RAR1*, *SGT1b* and *HSP90.2* by the root elongation assay (Figure S3). All *rar1* and *hsp90.2* mutants were as sensitive to MeJA treatment as wild type, suggesting that neither *RAR1* nor *HSP90.2*, plays a role in JA responses. As expected, the *sgt1b* mutant displayed an obvious insensitivity to MeJA [54]. We also noted MeJA insensitivity in the *rar1 sgt1b* double mutant (Figure S3). These results suggest that SGT1b is the only member of RAR1-SGT1-HSP90 NB–LRR co-chaperone complex required for JA signaling.

### *COI1* mutations do not affect the levels of RAR1, SGT1b, or HSP90 accumulation

*coi1* mutations restored the disease resistance responses mediated by three NB–LRR proteins in *rar1* (Figure 3A, Figure S2A and S2B) and thus possibly suppressed *rar1* via effects upon NB–LRR regulators that control the accumulation, and hence the function, of multiple NB–LRR proteins. To examine this possibility, we determined the accumulation levels of three NB–LRR regulators, RAR1 (Figure S5A), SGT1b (Figure S5B), and HSP90 (Figure S5C), in the *coi1^rsp^*, *coi1-1* and *coi1-16* mutants in either *RAR1* or *rar1* backgrounds. These *coi1* mutants did not exhibit any dramatic change of RAR1, SGT1b or HSP90 protein levels. Therefore, the *coi1^rsp^* and *coi1-16* alleles do not suppress *rar1* influencing by regulating the steady state levels of RAR1, SGT1b and/or HSP90.

### SGT1b antagonizes *coi1^rsp^*-mediated RPM1 accumulation and *RPM1*-dependent disease resistance in *rar1*

The *coi1^rsp^* mutants displayed opposite phenotypes: increased NB–LRR accumulation and function in *rar1* and decreased NB–LRR HR function in *RAR1*. A similar combination of phenotypes was previously observed in *sgt1b* as an *rar1* suppressor [25]. The *sgt1b* mutation enhanced RPS5 accumulation and consequent restoration of *RPS5*-mediated disease resistance in *rar1*, but did not restore *RPS5*-triggered HR in *RAR1* [25]. This similarity implies that *coi1^rsp^* mutants might regulate NB–LRR proteins by inhibiting the function of *SGT1b* and hence mimic *sgt1b* phenotypes.

**Figure 6:**
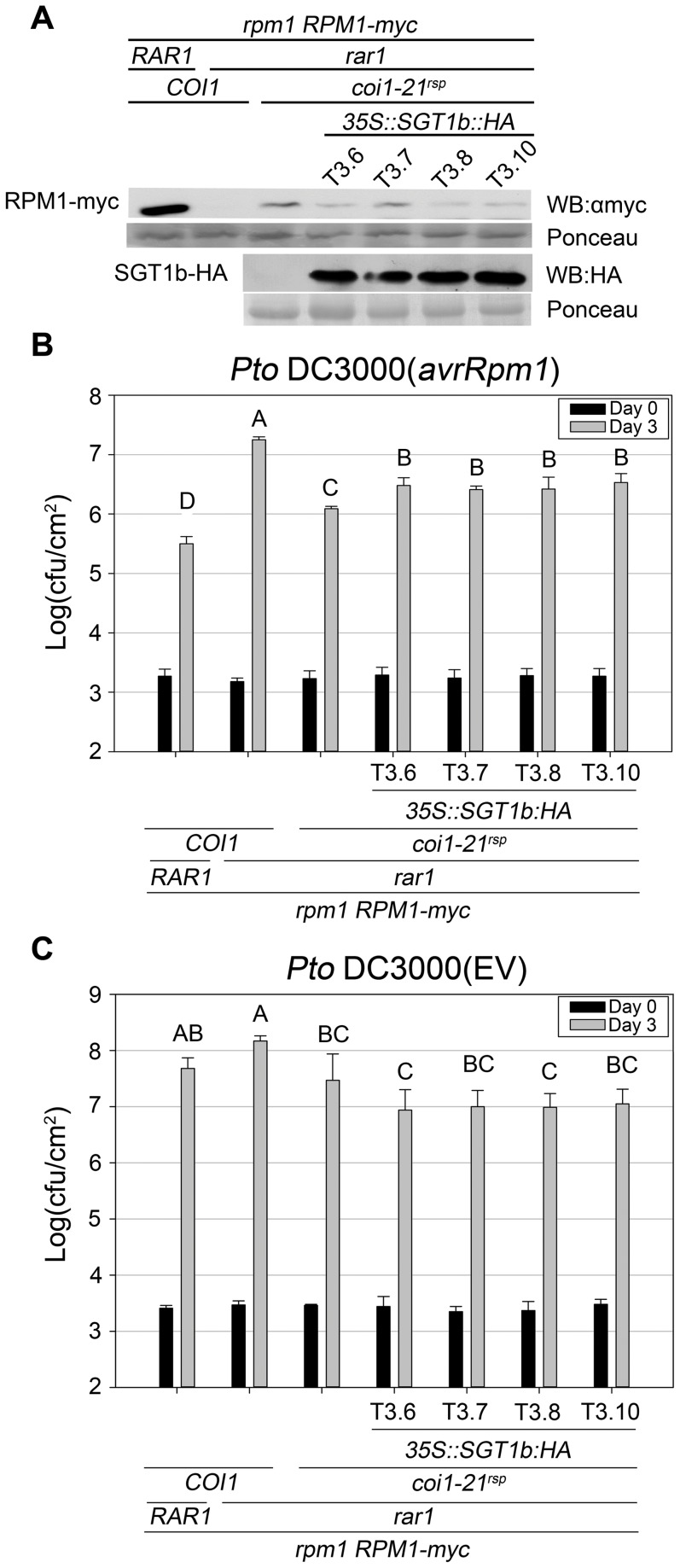
SGT1b over-expression antagonizes *coi1^rsp^*-dependent RPM1 accumulation and *RPM1*-mediated disease resistance in *rar1*. (A) Western blot analysis of RPM1-myc and SGT1b-HA protein levels in indicated genotypes. RuBisCo levels stained by Ponceau S serve as loading control. The result displayed is one of three independent blots giving similar results. (B–C) Bacterial growth analysis of *Pto* DC3000(*avrRpm1*) (B) and *Pto* DC3000(EV) (C). Bacteria were hand-infiltrated into leaves of each indicated genotype and counted at day 0 and day 3. Error bars represent 2× SE. Pair-wise comparisons for all means for bacterial growth on day 3 were performed with One-Way ANOVA test followed by Tukey-Kramer HSD at 95% confidence limits. The bacterial growth assays were performed independently three times (*Pto* DC3000(*avrRpm1*)) and twice (*Pto* DC3000(EV)) with similar results.

Based on this hypothesis, we expected that a high dose of SGT1b would attenuate the *rar1* suppression phenotypes of the *coi1^rsp^* mutants. To test this, we introduced a *35S:SGT1b-HA* construct into *coi1-21^rsp^ rar1* plants containing *RPM1-myc*. Compared with parental *coi1-21^rsp^ rar1* plants, four independent T3 lines that expressed relatively high levels of SGT1b::HA exhibited both reduced RPM1-myc levels (Figure 6A) and *RPM1*-mediated disease resistance (Figure 6B). However, the RPM1 accumulation and *RPM1*-mediated disease resistance observed in these T3 plants were still much higher than *rar1* plants (Figure 6A, 6B). These results demonstrated that modest over-expression of *SGT1b* can partially inhibit the *rar1* suppression phenotypes of *coi1^rsp^* alleles. As a control, we measured the growth of *Pto* DC3000(EV) in the plants used in the *Pto* DC3000(*avrRpm1*) growth assay. No enhanced growth of *Pto* DC3000(EV) was observed in these T3 lines (Figure 6C), demonstrating that the reduction of *RPM1*-mediated disease resistance in *35S:SGT1b-HA* transgenic plants are not due to a decrease in basal defense.

In addition, we measured the HSP90 protein levels and *RPM1-myc* mRNA levels in the transgenic plants used in the western blot analysis. No obvious decrease of HSP90 protein level (Figure S6A) or *RPM1-myc* mRNA level was detected (Figure S6B), indicating that the reductions of RPM1-myc accumulation in *35S:SGT1b-HA* transgenic plants are not due to the decrease of HSP90 accumulation or the silencing of *RPM1-myc* gene.

## Discussion

We initially performed a suppressor screen for mutants that could recover the diminished NB–LRR *RPS5*-mediated disease resistance phenotype of *rar1* [21]. These suppressors were isolated in the null *rar1-21* background (Figure S5A), and thus likely represent mutations that either bypass or counteract *rar1*. We reported two novel *HSP90* alleles derived from this screen that function to mimic the effects of RAR1 on the HSP90 lid open/close cycle required to stabilize NB–LRR clients [21]. Here, we detail the characterization of two *coi1* alleles, *coi1-21^rsp^* and *coi1-22^rsp^* also identified in this screen (Figure 1), and we note that a third single allele locus defined by *rsp3* has characteristics that suggest it might encode another new player in the regulation of NB–LRR accumulation (Figure S1). Because *rsp3* is a single, dominant allele, its description beyond the mutant phenotype was not pursued as part of this study.

The F-box protein COI1 is a core component of the receptor complex for jasmonate (JA) [28], [29], [31]. In plants, mutations in *COI1* impair all known JA responses and thus result in insensitivity to JA or functional JA derivatives [32], [33], [37], [43], [45]. As expected, both of the *coi1^rsp^* alleles were JA insensitive (Figure 5). However, the MeJA insensitivity in *coi1^rsp^* alleles is obviously weaker than in the null allele, *coi1-1* (Figure 5B).

In addition to being insensitive to JA, all *coi1* alleles identified previously are, at least partially, male-sterile [32], [33], [37], [43], [45]. To our surprise, the two *coi1^rsp^* alleles are completely fertile. Among the previously described alleles, only *coi1-8* (encoding a missense change of E543K) exhibits partial fertility in regular growth conditions [43]. The other partially fertile allele, *coi1-16* (encoding L245F), is fertile only at low temperature (16 degrees C) [45]. Similar to the *coi1^rsp^* alleles, *coi1-8* exhibited drastically reduced but still detectable COI1 protein levels [43]. A pull-down assay demonstrated that the COI1-8_E543K_ protein retains interaction with JAZ1, a substrate of COI1 in SCF^COI1^-mediated protein degradation [43]. These results indicate that the weak MeJA insensitivity and intact fertility of the *coi1^rsp^* mutants are likely due to lower accumulation of functional COI1^rsp^ proteins in these mutants. In other words, the *coi1^rsp^* mutations, G330E and G434E, cause relatively weaker impairments of the COI1 protein stability and activity than the other reported *coi1* missense alleles.

### COI1 functions in both basal defense and ETI

Mutations in *COI1* affect, negatively or positively, disease resistance against various plant pathogens [29], [33]–[41]. It is widely accepted that the defense phenotypes of *coi1* depend on signaling antagonism between SA and JA signaling pathways [55]. *COI1* mutations disable JA-signaling and consequently enhance SA signaling and SA-induced defense responses by an as yet unknown mechanism.

In Arabidopsis, resistance against the virulent hemi-biotrophic pathogen *Pto* DC3000 is a measure of basal defense [56]. In our study, all four tested *coi1* alleles, *coi1-21^rsp^*, *coi1-22^rsp^*, *coi1-1*, and *coi1-16* displayed enhanced disease resistance against *Pto* DC3000(EV) in both *rar1* and *RAR1* backgrounds (Figure 3B, Figure 4A). These results correspond to previously published data [29], [37], [38], and confirm that COI1 represses basal defense, likely via JA-SA antagonism. Besides enhanced basal defense, the *coi1* alleles also displayed enhanced ETI against *Pto* DC3000(*avrRpm1*) (Figure 3A, Figure 4B). Hence, COI1 also inhibits ETI. Since the enhancement of ETI was found in *rar1* mutant plants, RAR1, which is necessary for NB–LRR-mediated ETI in this and many other cases, is not required by COI1 to repress ETI.

### A plausible mechanism explaining COI1 effects on NB–LRR accumulation in *rar1* and *RAR1*

Although all four *coi1* alleles we analyzed restored resistance against *Pto* DC3000(*avrRpm1*) in *rar1* (Figure 3A), we could classify them into three classes based on how they influence RPM1 accumulation and *RPM1*-mediated immune response (Figure 3C, Figure 4C). Class I, represented by the null allele *coi1-1*, does not alter RPM1 levels. Class II, represented by *coi1-16*, enhances RPM1 levels in *rar1* and has no effect on *RPM1*-mediated HR in *RAR1*. Class III, represented by *coi1-21^rsp^* and *coi1-22^rsp^*, enhance RPM1 levels in *rar1*, but reduce *RPM1*-mediated HR in *RAR1*. Since the null *coi1-1* does not exhibit any detectable effect on RPM1 accumulation, the enhancement of RPM1 levels in *rar1* is a gain-of-function phenotype conferred by the COI1 mutant proteins accumulating in *coi1-16* and the two *coi1^rsp^* alleles. However, these alleles are all recessive for JA response phenotypes. The coexistence of these distinct genetic characteristics demonstrates that *coi1-16* and *coi1^rsp^* alleles are recessive gain-of-function alleles which have lost the JA signaling function of COI1, but gained new function, likely via interfering with the activity of other protein(s). RPM1 is associated with, and activated at, the plasma membrane; there is no current evidence suggesting that it shuttles into the nucleus [50], [57]. COI1 is expected to be localized in the nucleus, because it binds to the nucleus-localized JAZ proteins [58]. A biochemical mechanism to explain our genetic results would require a reconciliation of these findings. There may be sufficient coi1^rsp^ protein at the plasma membrane to mediate the effects on RPM1 that we describe. Further, our inference that COI1 has a wild type function in mediating NB–LRR protein accumulation is consistent with suggestions that nucleo-cytoplasmic shuttling is required for the function of at least a subset of NB–LRR proteins [3].

Some publications suggest that the “target” protein with which recessive gain-of-function alleles interfere can share functional redundancy with it [14], [59]–[62]. We found that mutants of two NB–LRR co-chaperones, SGT1b and HSP90, have phenotypic similarities with *coi1^rsp^* alleles [14], [21], [25]. These include (Table S3): 1) enhanced NB–LRR accumulation in *rar1*: RPM1 in *hsp90.2^rsp^ rar1* [21], RPS5 in *sgt1b rar1* [25], and RPM1 in *coi1^rsp^ rar1* (this work); 2) impaired NB–LRR-mediated HR in *RAR1*: *RPM1*-mediated HR in *hsp90^lra^* [14], *RPS5*-mediated HR in *sgt1b* [25], and *RPM1*-mediated HR in *coi1^rsp^* (this work). COI1 is an F-box protein which is a component of an SCF complex. Both SGT1b and HSP90 have been reported to associate and function with various SCF complexes in plants [13], [54], [63], [64]. These findings collectively imply that SGT1b and/or HSP90 are candidate target proteins of coi1^rsp^ proteins in suppressing *rar1*.

Since the *coi1^rsp^* alleles did not affect steady state SGT1b levels (Figure S5B), *coi1^rsp^* alleles might inhibit SGT1b activity to suppress the *rar1* phenotype of reduced NB–LRR accumulation. To test this hypothesis, we overexpressed SGT1b in a *coi1-21^rsp^ rar1* background. The *rar1* suppression phenotypes of *coi1-21^rsp^*, restored RPM1-myc accumulation and *RPM1*-mediated disease resistance, were partially complemented by SGT1b overexpression (Figure 6A, 6B). This result supports our hypothesis, and suggests that SGT1 functions with COI1 to regulate NB–LRR accumulation. On the other hand, the incomplete complementation could mean that we need higher levels of SGT1b over-expression, or that coi1^rsp^ proteins also down-regulate the activity of other targets, such as HSP90. Our speculation is supported by the non-allelic non-complementation observed between *coi1^rsp^* mutants and *hsp90.2-7^rsp^* mutant (Figure 2, Table S2). This specific genetic relationship suggests that COI1 and HSP90 physically interact with each other or belong to the same protein complex.

The RAR1-SGT1-HSP90 chaperone complex has been related to the SCF complex by two sorts of evidence: 1) SGT1b and HSP90 associate and function with various SCF complexes [13], [54], [63], [64]. RAR1 associates with the COP9 signalosome (CSN) which can inactivate the SCF complex [13], [64], [65]; 2) The SCF^CPR1^ complex negatively regulates the pre-activation steady state stability of two NB–LRR proteins, SNC1 and RPS2, via the F-box protein CPR1 [66]. The SCF component SKP1 is required for NB–LRR N protein-mediated resistance response against tobacco mosaic virus (TMV) [64]. This relationship suggests that RAR1-SGT1-HSP90 chaperone complexes function with an SCF-mediated protein degradation pathway to control the accumulation levels of NB–LRR protein and thus avoid inappropriate NB–LRR activation [19]. The phenotypes observed in our recessive gain-of-function *coi1^rsp^* mutants support this hypothesis. The *coi1^rsp^* mutants suppressed the *rar1* mutant for reduced NB–LRR RPM1 accumulation, and showed non-allelic non-complementation with *hsp90.2*. Moreover, overexpression of SGT1b partially inhibited the phenotypes of the *coi1^rsp^* mutants. Similar to *sgt1b* and *hsp90.2^lra^* mutants, *coi1^rsp^* mutants caused impaired HR function when moved to a wild type background. The sum of these results is consistent the idea that the F-box protein COI1 functions with RAR1-SGT1-HSP90 chaperone complex and consequently affects NB–LRR protein accumulation and function.

## Materials and Methods

### Plant lines

We used *coi1-1* [32] and *coi1-16* [45] as reference alleles. For the pathology analyses and root elongation analyses, mutant lines used (all in Col-0 background) were *rar1-21* [18], *rpm1-1* [67], *rps5-2* [68], *rps2-101c* [69], *sgt1b^edm1-1^* [17], *rar1-21 sgt1b^edm1-1^* [25], *hsp90.2-2* [14], *hsp90.2-5^KO^* [14], *hsp90.2-7* [21] and *hsp90.2-8* [21]. Ecotype Ws was used as an *rpp4* control [48]. We constructed *coi1-1 rar1-21* and *coi1-16 rar-21* double mutants by identifying F2s with PCR-based dCAP markers. The F2s with appropriate genotypes were selfed, and F3 individuals were further selected with PCR-based dCAP markers.

To make the *35S:SGT1b-HA* construct, the coding sequence of *SGT1b* without its stop-codon was amplified by PCR, and then moved into pGWB14 vector [70]. The final destination vector, pGWB14/35S:SGT1b-HA was electropolated into the Agrobacterium strain GV3101 for transformation of appropriate genotypes. Transformed plants were selected on MS medium plate (PhytoTechnology Laboratories, KS, U.S.) containing Hygromycin B (SIGMA, St. Louis, MO, U.S.).

### Pathogen strains, inoculation, growth quantification, and ion leakage assay

*Pto* DC3000 derivatives containing pVSP61(EV), *avrRpm1*, *avrPphB*, and *avrRpt2* were maintained as described [71]. Plant inoculations and bacterial growth assays were performed as described (spray-inoculation [21]; dip-inoculation [72]; hand-inoculation [25]). The HR test and ion leakage assays were carried out as described [21].

*Hyaloperonospora arabidopsidis* (*Hpa*) isolate Emwa1 was used to inoculated ten-day-old cotyledons of plants as described [21]. Asexual sporangiophores were counted 7 days post-inoculation on at least 30 cotyledons for each genotype.

### Identification and map-based cloning of mutations in *COI1*

The *rar1* suppressor screen was previously described [21]. Standard genetic analyses and map-based cloning were performed as described [21]. We used 892 disease resistant F_2_ individuals to define a 60 Kb interval on the chromosome II containing *COI1*. By sequencing *COI1* in the originally isolated *rar1* suppressor mutant, a G/A transition at position 1849 (nucleotide positions relative to the translation start site of the published sequence of *COI1*; AT2G39940) was identified in *coi1-21^rsp^*. The other mutant, *coi1-22^rsp^*, also contains a G/A mutation at position 2161 in *COI1*. To obtain *coi1-21^rsp^* and *coi1-22^rsp^* single mutants, we backcrossed the *coi1^rsp^* alleles into an isogenic *RAR1* background. PCR-based dCAP markers were designed for selecting these two *coi1^rsp^* mutations.

### MeJA treatment

For growth inhibition assays, seedlings were grown on MS medium with different concentrations of Methyl Jasmonate (MeJA) (SIGMA) at 22°C under 16 h light/8 h dark photoperiod. 10-day-old seedlings were taken picture to show the inhibition effects.

For root elongation assays, seedlings were horizontally grown on MS medium at 22°C under 24 h light for 4 d. Then seedlings were transferred to new MS medium with or without 50 µM MeJA, and grown for additional 4 d. Root elongations during these four days were measured.

### Western blots

For detection of RPM1-myc in the genotypes mentioned in this study, we introduced by crossing and segregation the mutants into plants expressing *RPM1-myc* from the native *RPM1* promoter as described [14]. The protein extraction and western blot were performed as described [14]. For detection of SGT1b-HA in plants, the protein extraction and western blot were carried out based on the protocol that was previously used for RPS5-HA [25]. The anti-COI1 antiserum was kindly provided by Daoxin Xie (Tsinghua University, Beijing, China). The protein extraction and western blot were performed as described [42]. anti-SGT1 and anti-RAR1 polyclonal antibodies against the full length SGT1b and full length RAR1 with C-terminus GST tag were generated in rabbits (custom products of Cocalico Biologicals, Inc.). anti-HSP90-2 was the product of Agrisera company (Swedish). The detailed protocols for detection of SGT1a, SGT1b, RAR1, and HSP90 proteins are provided as Text S1.

### RT–qPCR

Plant RNA was extracted with RNeasy Plant Mini Kit (Qiagen). To eliminate DNA contamination, RNA was purified by Turbo DNA Free Kit (Ambion) and RNeasy Mini Kit (Qiagen). 2 µg RNA was reverse transcribed with Random Decamers and RETROscript kit (Ambion).

RT-qPCR was performed in a total volume of 25 µl (12.5 µl SYBR Green PCR Master Mix (Applied Biosystems), 0.5 µl cDNA, 1 µl Primer 1 (10 µM), 1 µl Primer 2 (10 µM) and 10 µl H_2_O) with MJ White 96-well plate and a DNA Engine OPTICON 2 system (MJ Research). The reaction was run at 95°C for 5 min, followed by 40 cycles at 95°C for 15 sec, 55°C for 30 sec and 72°C for 30 sec. Dissociation analysis was performed after each reaction to confirm the specificity. The relative expression of *RPM1/RPM1-myc* gene in different genotypes was calculated by ΔΔCt method (User Bulletin #2, Manual of Applied Biosystems). The primers were newly designed or obtained from previous publication [73], and are provided as Text S1.

## Supporting Information

Figure S1 *rsp3* can suppress all known *rar1* phenotypes. In addition to two *hsp90.2^rsp^* and two *coi1^rsp^* mutants, we isolated a fifth mutant, named *rsp3* (*rar1 suppressor 3*), from the *rar1* suppressor screen. Based on disease symptoms after inoculation with *Pto* DC3000(*avrRpm1*), we determined that *rsp3* is dominant. The results of bacterial growth experiments showed that the *rsp3* mutant suppressed *rar1* for disease resistance functions of *RPS5* (Figure S1A), *RPM1* (Figure S1B) and *RPS2* (Figure S1C). *rsp3* also fully suppressed the decreased basal disease resistance phenotype of *rar1* (Figure S1D). In addition to disease resistance, *RPM1*-mediated HR in *rar1* was also suppressed by *rsp3* (Figure S1E). We also noted that *rsp3* partially suppressed the loss of *RPP4* function in *rar1* following infection with *Hpa* Emwa1 (Figure S1F). Finally, we also found that *rsp3* mutant suppressed the lowered accumulation of RPM1 in *rar1* (Figure S1G). The combined phenotypes of *rsp3 rar1* mutant demonstrated that *rsp3* suppressed, fully or partially, all known *rar1* phenotypes. Using map-based cloning, we localized *rsp3* mutation in a 7 Mbp (from 15.9 Mbp to 22.9 Mbp) mapping interval on chromosome I (Figure S1H). No NB–LRR regulator has been found in this interval. However, because it is a single dominant allele, further characterization of *rsp3* is beyond the scope of this paper. (A–B) Bacterial growth assays of (A) *Pto* DC3000(*avrPphB*), (B) *Pto* DC3000(*avrRpm1*), (C) *Pto* DC3000(*avrRpt2*) and (D) *Pto* DC3000(EV). Leaves of each indicated genotype were dip-inoculated [1].Bacteria were counted at day 0 and day 3. Error bars represent 2× SE; (E) Conductivity measurements after inoculation with high concentration of *Pto* DC3000(*avrRpm1*) (5×10^7^ cfu/ml). Error bars represent 2×SE; (F) 10-day-old cotyledons were inoculated with *Hpa* isolate Emwa1. Asexual sprangiophores were quantified 7 days after inoculation on cotyledons for each of the indicated genotypes [2]. (Sp: sprangiophore); (G) Western blot analysis of RPM1-myc protein levels in indicated genotypes. RuBisCo levels stained by Ponceau S serve as loading control; (H) The positions of the Simple Sequence Length Polymorphisms (SSLP) markers used for rough map-based cloning on chromosome I. *rsp3* mutation was localized in the mapping interval between marker T27K12(15.9 Mbp) and F19K23(22.9 Mbp). The pathogen growth and HR assays were performed independently a minimum of three times with similar results. The RPM1-myc blot displayed is one of three independent blots giving similar results.
(TIF)

Figure S2 *coi1^rsp^* alleles suppress some, but not all *rar1* phenotypes for NB–LRR function. (A–B) Bacterial growth analysis of *Pto* DC3000(*avrPphB*) (A) and *Pto* DC3000(*avrRpt2*) (B) Leaves of indicated genotypes were dip-inoculated [1]. Bacteria were counted for day 0 and day 3. Error bars represent 2× SE. Pairwise comparisons for all means for bacterial growth on day 3 were performed with One-Way ANOVA test followed by Tukey-Kramer HSD at 95% confidence limits; (C) Conductivity measurements after inoculation with high concentration *Pto* DC3000(*avrRpm1*) (5×10^7^ cfu/ml). Error bars represent 2× SE; (D) 10-day-old cotyledons were inoculated with *Hpa* isolate Emwa1. Asexual sprangiophores were quantified 7 days after inoculation on cotyledons for each of the indicated genotypes [2]. (Sp: sprangiophore). The pathogen growth and HR assays were performed independently a minimum of three times with similar results.
(TIF)

Figure S3 *coi1^rsp^* alleles and *sgt1b* are insensitive to MeJA. Inhibition of root elongation by 50 µM MeJA for the indicated genotypes. The root elongation assay was performed three times with similar results. At least fifteen seedlings per genotype were measured in each repeat. Error bar represents 2×SE.
(TIF)

Figure S4 *coi1^rsp^* and *coi1-16* mutations do not enhance *RPM1* transcript levels. RT-qPCR analysis of the expression of *RPM1* for indicated genotypes. The result displayed is one of three independent RT-qPCRs giving similar results.
(TIF)

Figure S5 *COI1* mutants studied express wild type levels of RAR1, SGT1 and HSP90 proteins. Western blot analysis of SGT1b, SGT1a, RAR1 and HSP90 protein levels for the indicated genotypes. RuBisCo levels stained by Ponceau S serve as loading control. The western blots were performed twice independently with similar results.
(TIF)

Figure S6 The reductions of RPM1-myc levels in the *35S:SGT1b-HA* transgenic plants are not due to the decrease of HSP90 protein level or the silencing of *RPM1-myc* gene. (A) Western blot analysis of RPM1-myc, SGT1b, SGT1a, and HSP90 protein levels for the indicated genotypes. RuBisCo levels stained by Coomassie Brilliant Blue serve as loading control; (B) RT-qPCR analysis of the expression of *RPM1* and *RPM1-myc* for the indicated genotypes. The western blot and RT-qPCR assay were performed independently a minimum of two times with similar results.
(TIF)

Table S1 Both of the *coi1^rsp^* mutants are completely recessive.
(DOC)

Table S2 Non-allelic non-complementation between *coi1^rsp^* and *hsp90^rsp^* mutants. RPM1-mediated resistance was tested by spray-inoculation with *Pto* DC3000(*avrRpm1*). Disease symptoms were evaluated 5 days after inoculation.
(DOC)

Table S3 Phenotypic similarities among *coi1*, *sgt1b*, and *hsp90.2* mutants.
(DOC)

Text S1 Supporting information including the primers and corresponding enzymes for selecting of mutations, the primers for making *35S::SGT1b-HA* construct, the primers used for RT-qPCR, and additional information of Western blots.
(DOC)
