# Supplementary figures and images for: Specific Missense Alleles of the Arabidopsis Jasmonic Acid Co-Receptor COI1 Regulate Innate Immune Receptor Accumulation and Function
Source: PLoS Genet. 2012 Oct 18;8(10):e1003018. doi: 10.1371/journal.pgen.1003018 (PMC3475666; doi:10.1371/journal.pgen.1003018)

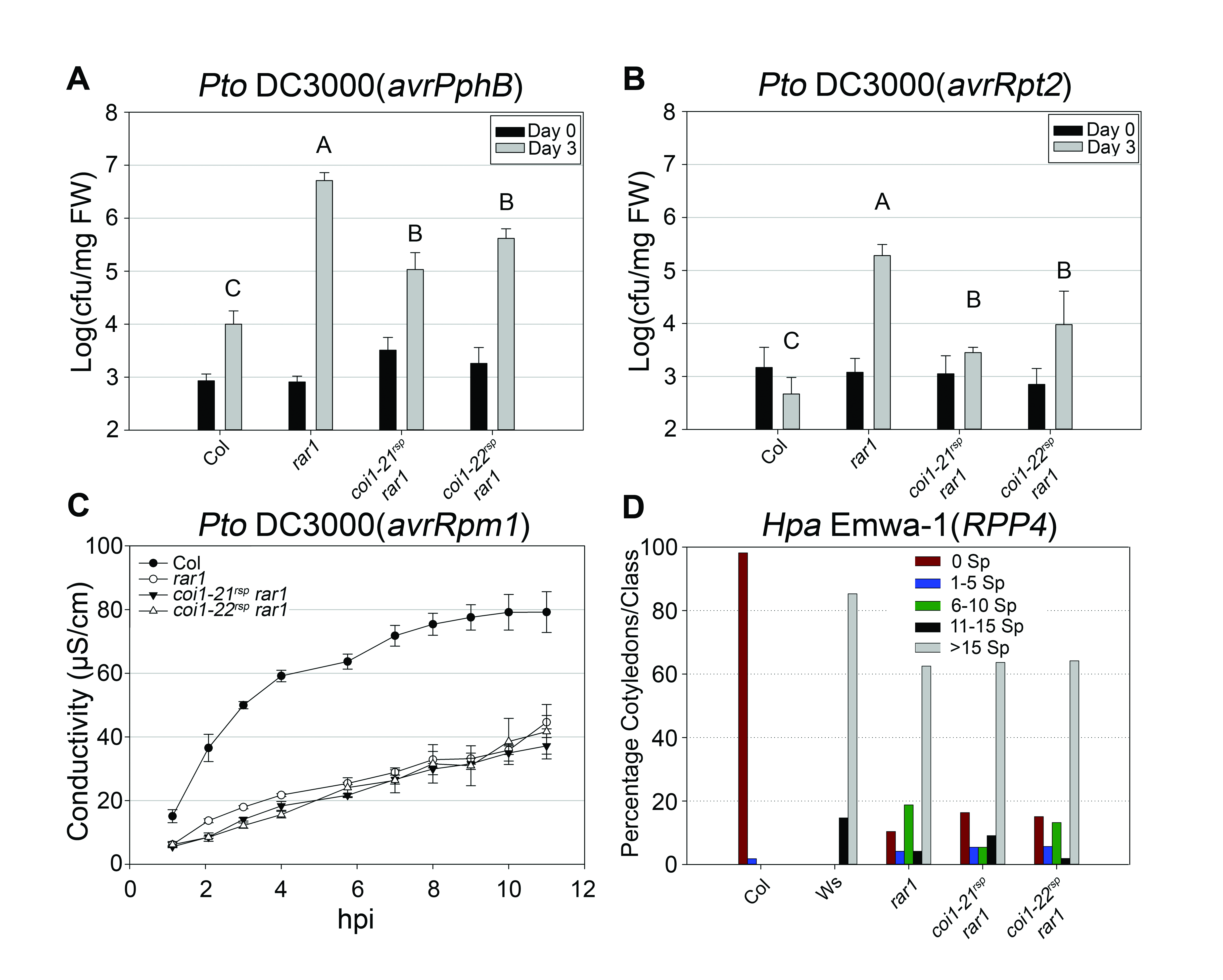

Supplement: Figure S2 — coi1rsp alleles suppress some, but not all rar1 phenotypes for NB–LRR function. (A–B) Bacterial growth analysis of Pto DC3000(avrPphB) (A) and Pto DC3000(avrRpt2) (B) Leaves of indicated genotypes were dip-inoculated [1]. Bacteria were counted for day 0 and day 3. Error bars represent 2× SE. Pairwise comparisons for all means for bacterial growth on day 3 were performed with One-Way ANOVA test followed by Tukey-Kramer HSD at 95% confidence limits; (C) Conductivity measurements after inoculation with high concentration Pto DC3000(avrRpm1) (5×10^7^ cfu/ml). Error bars represent 2× SE; (D) 10-day-old cotyledons were inoculated with Hpa isolate Emwa1. Asexual sprangiophores were quantified 7 days after inoculation on cotyledons for each of the indicated genotypes [2]. (Sp: sprangiophore). The pathogen growth and HR assays were performed independently a minimum of three times with similar results. (TIF) [file pgen.1003018.s002.tif]

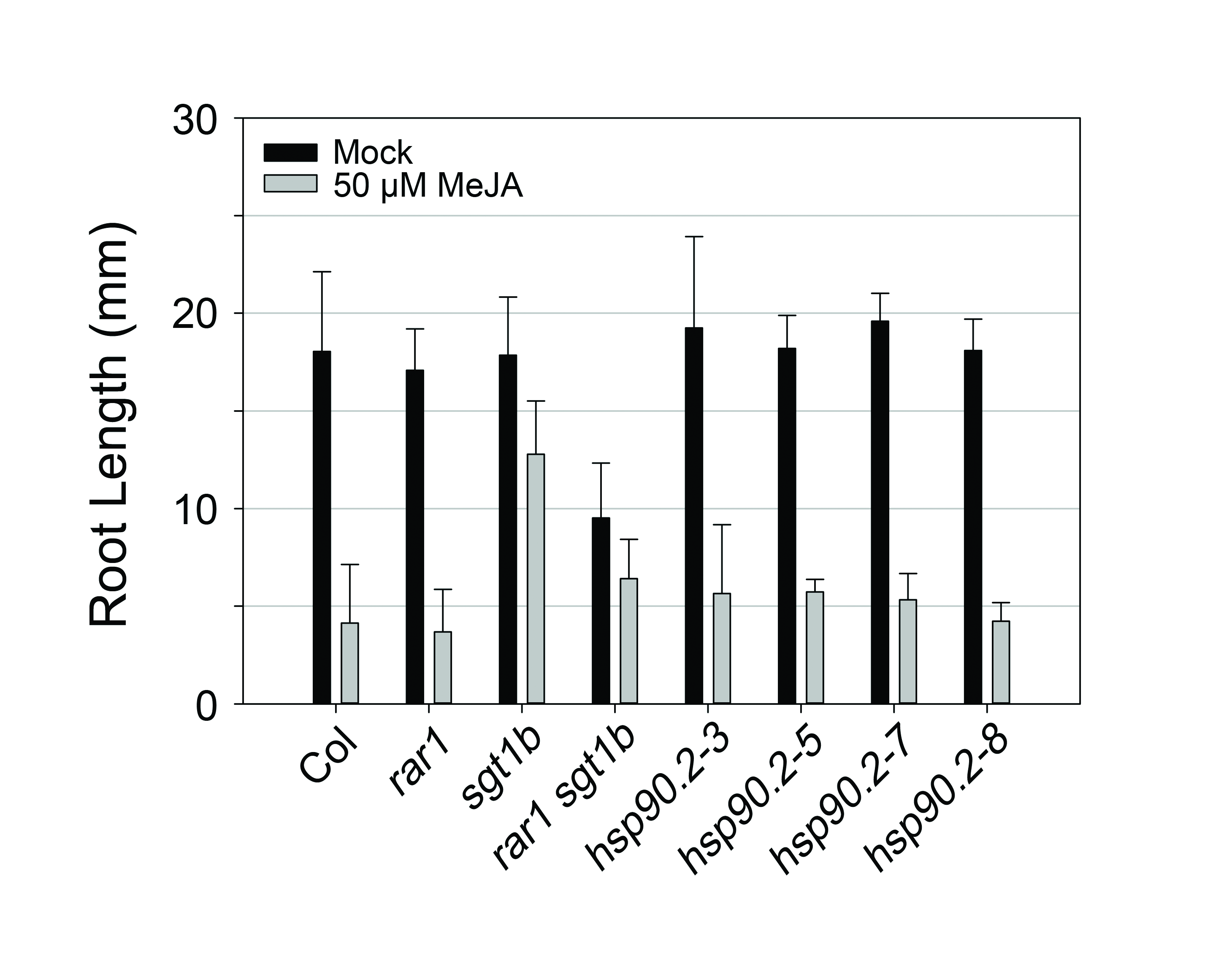

Supplement: Figure S3 — coi1rsp alleles and sgt1b are insensitive to MeJA. Inhibition of root elongation by 50 µM MeJA for the indicated genotypes. The root elongation assay was performed three times with similar results. At least fifteen seedlings per genotype were measured in each repeat. Error bar represents 2×SE. (TIF) [file pgen.1003018.s003.tif]

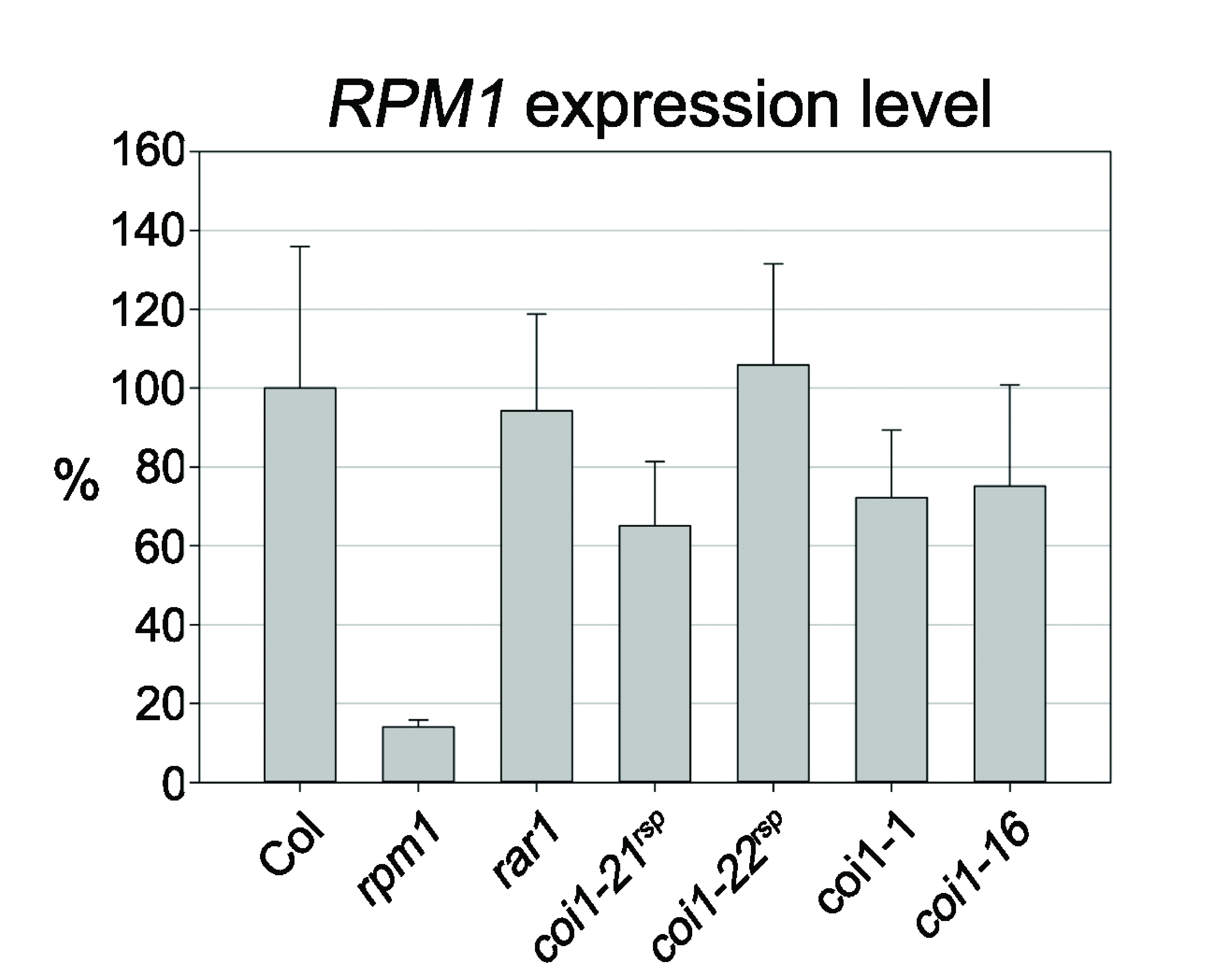

Supplement: Figure S4 — coi1rsp and coi1-16 mutations do not enhance RPM1 transcript levels. RT-qPCR analysis of the expression of RPM1 for indicated genotypes. The result displayed is one of three independent RT-qPCRs giving similar results. (TIF) [file pgen.1003018.s004.tif]

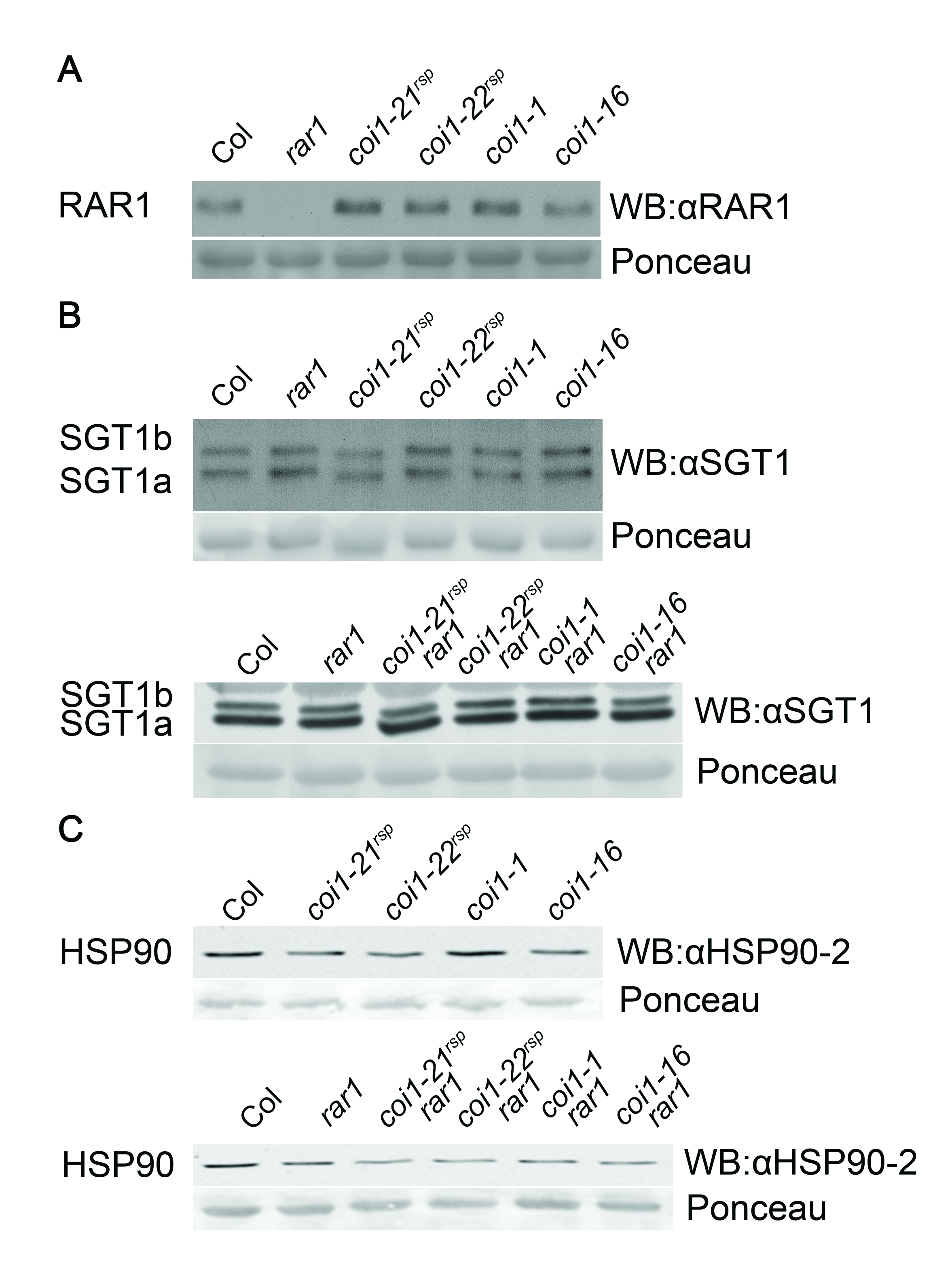

Supplement: Figure S5 — COI1 mutants studied express wild type levels of RAR1, SGT1 and HSP90 proteins. Western blot analysis of SGT1b, SGT1a, RAR1 and HSP90 protein levels for the indicated genotypes. RuBisCo levels stained by Ponceau S serve as loading control. The western blots were performed twice independently with similar results. (TIF) [file pgen.1003018.s005.tif]

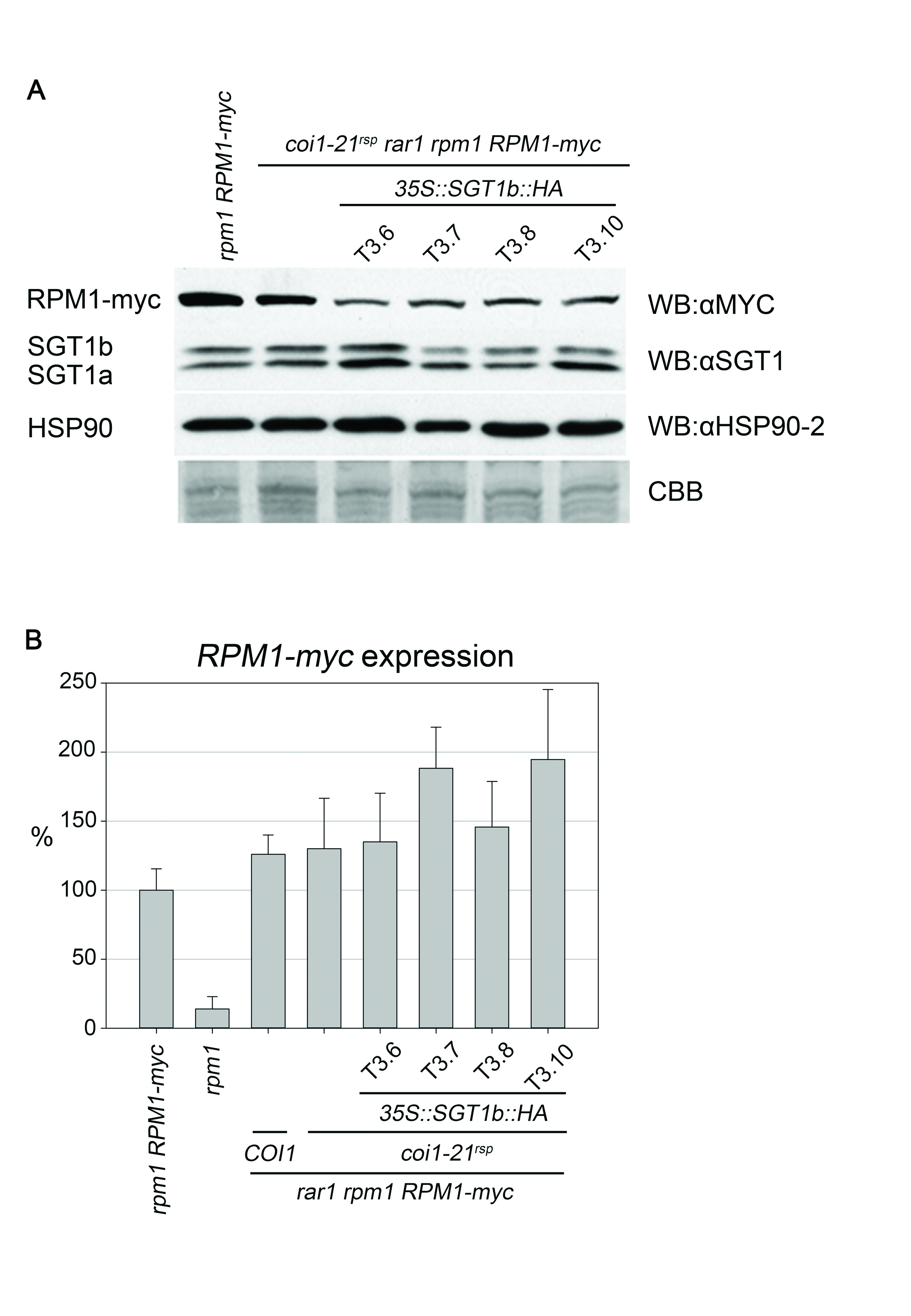

Supplement: Figure S6 — The reductions of RPM1-myc levels in the 35S:SGT1b-HA transgenic plants are not due to the decrease of HSP90 protein level or the silencing of RPM1-myc gene. (A) Western blot analysis of RPM1-myc, SGT1b, SGT1a, and HSP90 protein levels for the indicated genotypes. RuBisCo levels stained by Coomassie Brilliant Blue serve as loading control; (B) RT-qPCR analysis of the expression of RPM1 and RPM1-myc for the indicated genotypes. The western blot and RT-qPCR assay were performed independently a minimum of two times with similar results. (TIF) [file pgen.1003018.s006.tif]
