# Supplementary material for: Specific Missense Alleles of the Arabidopsis Jasmonic Acid Co-Receptor COI1 Regulate Innate Immune Receptor Accumulation and Function
Source: PLoS Genet. 2012 Oct 18;8(10):e1003018. doi: 10.1371/journal.pgen.1003018 (PMC3475666; doi:10.1371/journal.pgen.1003018)
Supplement: Table S1 — Both of the coi1rsp mutants are completely recessive. (DOC) [file pgen.1003018.s007.doc]

**Table S1. Both of the *coi1^rsp^* mutants are completely recessive.**

| ***RPM1*-mediated resistance in plants** | |
| --- | --- |
|  | Number of plants exhibiting resistance/Total tested plants |
| *coi-21^rsp^ rar1* | 12/12 |
| *coi-22^rsp^ rar1* | 12/12 |
| *rar1* | 0/12 |
| *coi-21^rsp^ rar1* x *rar1* F1 | 0/8 |
| *coi-22^rsp^ rar1* x *rar1* F1 | 0/6 |
| *coi-21^rsp^ rar1* x *rar1* F2 | 31/108 |
| *coi-22^rsp^ rar1* x *rar1* F2 | 26/86 |
