# Supplementary material for: Specific Missense Alleles of the Arabidopsis Jasmonic Acid Co-Receptor COI1 Regulate Innate Immune Receptor Accumulation and Function
Source: PLoS Genet. 2012 Oct 18;8(10):e1003018. doi: 10.1371/journal.pgen.1003018 (PMC3475666; doi:10.1371/journal.pgen.1003018)
Supplement: Table S2 — Non-allelic non-complementation between coi1rsp and hsp90rsp mutants. RPM1-mediated resistance was tested by spray-inoculation with Pto DC3000(avrRpm1). Disease symptoms were evaluated 5 days after inoculation. (DOC) [file pgen.1003018.s008.doc]

**Table S2. Non-allelic non-complementation between *coi1^rsp^*and *hsp90^rsp^* mutants.**

| ***RPM1*-mediated resistance in plants** | |
| --- | --- |
|  | Number of plants exhibiting resistance/Total tested plants |
| *coi-21^rsp^ rar1* | 12/12 |
| *coi-22^rsp^ rar1* | 12/12 |
| *hsp90.2-7^rsp^ rar1* | 12/12 |
| *coi-21^rsp^ rar1*  x  *hsp90.2-7^rsp^ rar1*  F1 | 5/5 |
| *coi-22^rsp^ rar1*  x  *hsp90.2-7^rsp^ rar1*  F1 | 4/4 |
| *coi-21^rsp^ rar1*  x  *hsp90.2-7^rsp^ rar1*  F2 | 25/36 |
| *coi-22^rsp^ rar1*  x  *hsp90.2-7^rsp^ rar1*  F2 | 26/36 |
