# Supplementary material for: Specific Missense Alleles of the Arabidopsis Jasmonic Acid Co-Receptor COI1 Regulate Innate Immune Receptor Accumulation and Function
Source: PLoS Genet. 2012 Oct 18;8(10):e1003018. doi: 10.1371/journal.pgen.1003018 (PMC3475666; doi:10.1371/journal.pgen.1003018)
Supplement: Table S3 — Phenotypic similarities among coi1, sgt1b, and hsp90.2 mutants. (DOC) [file pgen.1003018.s009.doc]

**Table S3. Phenotypic similarities among *coi1*, *sgt1b*, and *hsp90.2* mutants [1-3].**

| mutant phenotype | *coi1^rsp^* | *sgt1b^edm1^* | *hsp90.2^rsp^* | *hsp90.2^lra^* |
| --- | --- | --- | --- | --- |
| NB-LRR-mediated resistance responses in *RAR1* | no phenotype | no phenotype | no phenotype | reduced RPM1-mediated resistance |
| NB-LRR-mediated HR in *RAR1* | reduced RPM1-mediated HR | reduced RPS5-mediated HR | no phenotype | reduced RPM1-mediated HR |
| NB-LRR-mediated resistance responses in *rar1* | enhanced RPM1, RPS2, and RPS5-mediated resistance | enhanced RPS5-mediated resistance | enhanced RPM1, RPS2, and RPS5-mediated resistance | no phenotype |
| NB-LRR-mediated HR in *rar1* | no phenotype | no phenotype | enhanced RPM1-mediated HR | no phenotype |
| NB-LRR accumulation in *rar1* | enhanced RPM1 accumulation | enhanced RPS5 accumulation | enhanced RPM1 accumulation | no phenotype |

*rsp*: *rar1 suppressor; lra: loss of recognition of avrRpm1*

**References**

1. Holt BF, 3rd, Belkhadir Y, Dangl JL (2005) Antagonistic control of disease resistance protein stability in the plant immune system. Science 309: 929-932.

2. Hubert DA, Tornero P, Belkhadir Y, Krishna P, Takahashi A, et al. (2003) Cytosolic HSP90 associates with and modulates the Arabidopsis RPM1 disease resistance protein. EMBO J 22: 5679-5689.

3. Hubert DA, He Y, McNulty BC, Tornero P, Dangl JL (2009) Specific Arabidopsis HSP90.2 alleles recapitulate RAR1 cochaperone function in plant NB-LRR disease resistance protein regulation. Proceedings of the National Academy of Sciences 106: 9556-9563.
