# Supplementary material for: Specific Missense Alleles of the Arabidopsis Jasmonic Acid Co-Receptor COI1 Regulate Innate Immune Receptor Accumulation and Function
Source: PLoS Genet. 2012 Oct 18;8(10):e1003018. doi: 10.1371/journal.pgen.1003018 (PMC3475666; doi:10.1371/journal.pgen.1003018)
Supplement: Text S1 — Supporting information including the primers and corresponding enzymes for selecting of mutations, the primers for making 35S::SGT1b-HA construct, the primers used for RT-qPCR, and additional information of Western blots. (DOC) [file pgen.1003018.s010.doc]

**Materials and Methods S1**

**The primers and corresponding enzymes for selecting of mutations**

*coi1-1*: 5’-GGC GGT GTA TGT CTC AGA TAT AAC TAA CGA ATC TCT TGA AAG C-3’ and 3’-CCT TCA TCT GAT TCA CCT ACG TAA CCC AGC AGA AT-5’ (*Eco*RI);

*coi1-16*: 5’-GAG ATT TTG GAA CTA GTT GGG TTC TTT AAG GCT GCA GCG AAT-3’ and 3’- CAG CAA AGT TAA ACG TTT ACC TCG AGA ACT TCC AAA TTA GGA C-5’ (*Hin*fI);

*rar1-21*: 5’-TCA CGA CGG AAT GAA AGA GTG GAG CTG CTA CTA G-3’ and 3’-TTT TGG AAC CGA TTT GGC CAG AAC TGG TTT CTC AG-5’ (*Spe*I);

*coi1-21^rsp^*: 5’- GAC AAC ACT TGT TGT TTT TCT TCA GAC AAG GAA TGT AAC CG-3’ and 3’-GGT CGA GTA AGA CAA GGC GGA AGT CAC AGA GGT T-5’ (*Hpa*II);

*coi1-22^rsp^*: 5’-CTG TAA GCA GTT GAA GCG GCT GAG GAT TGA A-3’ and 5’-GTC TCA GAT AGA ATG CAA ATC GTC TGA GTT TCT TGG AT-5’ (*Bam*HI).

**The primers for making *35S::SGT1b-HA* construct**

5’-CAC CAT GGC CAA GGA ATT AGC AGA GAA AGC TAA AGA AGC T-3’

3’-ATA CTC CCA CTT CTT GAG CTC CAT GCC ATC TG-5’.

**The primers used for RT-qPCR**

*RPM1/RPM1-myc* gene: 5’-CGC CAT GAG CTT ACC AAA CAG ATT G-3’ and 3’- CAG TGG AAA CTG CTG TGA AGT GAG C-5’.

The reference gene *ELONGATION FACTOR 1* (*EF1*): 5’-CTG GAG GTT TTG AGG CTG GTA T-3’ and 3’-CCA AGG GTG AAA GCA AGA AGA-5’.

**Western Blots**

For detection of SGT1a, SGT1b, RAR1 and HSP90, protein was extracted by the method used for RPM1-myc. Equal loading of protein samples was performed by quantifying each sample with Bio-Rad Protein Assay (Bio-Rad, Hercules, CA, U.S.). Proteins were separated on 10% SDS-PAGE gel and transferred onto Amersham Hybond^TM^-P membranes (GE Healthcare). The blots were blocked in TBST (TBS with 0.05% Tween 20) containing 3% nonfat milk for 1 h and incubated for an additional 1 h with primary antibody (5000 times (anti-SGT1)/5000 times (anti-RAR1)/10000 times (anti-HSP90-2) diluted with TBST containing 1% nonfat milk) at room temperature. Then the blots were washed for 10 min two times with 1xTBST and incubated with secondary antibody goat-anti-rabbit IgG–HRP (GE Healthcare) (prepared in TBST containing 1% nonfat milk) for 1 h. IgG-HRP was detected with ECL Plus Western Blotting Detection System (GE Healthcare) after washing for 10 min three times with 1xTBST.
